# Diagnostic value of CT liver-to-spleen attenuation ratio in patients with non-alcoholic fatty liver disease and atherosclerotic plaque

**DOI:** 10.12669/pjms.40.10.10578

**Published:** 2024-11

**Authors:** Dong Han, Chongan He, Shuang Gu, Dongxuan Zhang, Liyun Xu

**Affiliations:** 1 Dong Han Department of Medical Imaging, Beijing Changping Hospital of Chinese Medicine, Beijing 102200, P.R. China; 2 Chongan He Department of Medical Imaging, Beijing Changping Hospital of Chinese Medicine, Beijing 102200, P.R. China; 3 Shuang Gu Department of Medical Imaging, Beijing Changping Hospital of Chinese Medicine, Beijing 102200, P.R. China; 4 Dongxuan Zhang Department of Spleen and Stomach Diseases, Beijing Changping Hospital of Chinese Medicine, Beijing 102200, P.R. China; 5 Liyun Xu Department of Ultrasound, Beijing Changping Hospital of Chinese Medicine, Beijing 102200, P.R. China

**Keywords:** CT Liver-to-spleen attenuation ratio, Non-alcoholic fatty liver disease, Atherosclerotic plaque

## Abstract

**Objective:**

To explore the clinical value of computed tomography (CT) liver-to-spleen (L/S) attenuation ratio in patients with non-alcoholic fatty liver disease (NAFLD) accompanied by atherosclerotic plaque (AP).

**Methods:**

This was a single-center, retrospective, observational study of patients who were diagnosed with NAFLD undergoing CT scans at Beijing Changping Hospital of Chinese Medicine from April 2020 to April 2022. Patients were grouped according to whether they had a diagnosis of AP or not. Healthy individuals without NAFLD undergoing CT scans during the same period were also included as a control group. The patients were matched for gender, age, and BMI in a 1:1:1 ratio. Correlations between the CT L/S attenuation ratio, liver function indicators, and blood lipid levels were assessed in the three groups. The predictive value of the CT L/S attenuation ratio was evaluated using the receiver operating characteristic (ROC) curve and the area under the curve (AUC) analyses.

**Results:**

Eighty-nine cases in each group. The three groups had significant differences in liver function and blood lipid levels (*P*<0.05). The CT L/S attenuation ratio in the NAFLD+AP and NAFLD groups was lower than that in the control group and was the lowest in the NAFLD+AP group (*P*<0.05). There was no significant correlation between the CT L/S attenuation ratio and liver function indicators (*P*>0.05), but it positively correlated with high-density lipoprotein (HDL) and negatively correlated with low-density lipoprotein (LDL), triglycerides (TG), and total cholesterol (TC) (*P*<0.05). The CT L/S attenuation ratio had a high predictive value for NAFLD patients with AP (AUC=0.859).

**Conclusions:**

The CT L/S attenuation ratio in NAFLD patients with AP is significantly reduced and is closely related to the levels of blood lipid indicators. The CT L/S attenuation ratio has a high predictive value for NAFLD patients with AP.

## INTRODUCTION

Non-alcoholic fatty liver disease (NAFLD) is a type of chronic liver disease that is characterized by varying degrees of fat accumulation in liver cells (steatosis) and is closely related to metabolic disorders and obesity.^1^,^2^ Numerous studies show a close relationship between NAFLD, hypertension, hyperlipidemia, Type-2 diabetes, and other metabolic syndromes and the development of atherosclerotic plaques (AP) and cardiovascular disease (CVD).^2^-^5^ In recent years, with lifestyle and dietary changes, the continuously rising incidence rate of NAFLD has become a public health problem, necessitating constant development of effective diagnostic methods.^6^,^7^

In clinical practice, color Doppler ultrasound is routinely used to diagnose and evaluate liver fat infiltration status and NAFLD+AP. However, its sensitivity and specificity often do not meet clinical expectations, leading to limitations in its clinical application.^8^-^10^ Computed tomography (CT) has gradually become more popular in the diagnosis and treatment evaluation of NAFLD.^11^ Current CT diagnostic criteria for steatosis are based on the ratio between liver and spleen attenuation,^12^ and the sensitivity and specificity of CT for steatosis currently range from 43 to 95% and 90–100%, respectively.^13^,^14^ A study by Poyraz et al.^15^ reported that the incidence of CVD was lower in patients with a high liver-to-spleen (L/S) ratio. However, there is still a lack of extensive research on the possible correlation between CT L/S attenuation ratio and the incidence of NAFLD accompanied by AP. This retrospective study aimed to evaluate the clinical value of CT L/S attenuation ratio in patients with NAFLD and AP.

## METHODS

This was a single-center, retrospective, observational study of patients who were diagnosed with NAFLD and underwent CT scans at Beijing Changping Hospital of Chinese Medicine from April 2020 to April 2022. Patients were grouped according to the presence of the AP diagnosis. Healthy individuals without NAFLD undergoing CT scans during the same period were also included as a control group. The patients were matched for gender, age, and BMI in a 1:1:1 ratio.

### Ethics Approval:

This study was approved by the Ethics Review Board of the Beijing Changping Hospital of Chinese Medicine (No. 2022-04-01; Date: 29-April, 2022).

### Inclusion criteria:

- Patients in the NAFLD+AP and the NAFLD groups were diagnosed with NAFLD.^16^
- AP diagnosis of patients in the NAFLD+AP group was confirmed by coronary computed tomography angiography (CTA) examination.^17^
- All patients underwent CT examination, and the liver-to-spleen attenuation ratios were recorded.
- The clinical data of the patients were complete.

### Exclusion criteria:

- Patients with hyperlipidemia, hypertension, or diabetes.
- Patients with poor contrast agent filling and excessive motion artifacts.
- Patients with history of alcohol consumption of over 140 g per week.
- Patients who were taking drugs that may affect liver metabolism within one month before inclusion in the study.
- Patients with malignant tumors; and women during lactation and pregnancy.

### Outcome measures:

- The CT L/S attenuation ratio: CT was performed by the same team of experienced physicians using a 16-row spiral CT from General Electric (GE). The largest slice was selected to measure the CT values of the spleen (S) and liver (L). The area of interest was over 1 cm^2^, and bile duct structure and vessels were avoided as much as possible. The right lobe of the liver and spleen on the same plane were measured twice, and the average value was calculated;
- Liver function and blood lipid index levels: 5 ml fasting venous blood was taken from each patient, added with anticoagulant, and replaced in EP tubes, centrifuged at 1000rpm for five minutes at 4°C, and the supernatant was taken. Levels of liver function indicators alanine aminotransferase (ALT), aspartate aminotransferase (AST), and gamma-glutamyltransferase (GGT), as well as levels of blood lipid indicators (high-density lipoprotein (HDL), low-density lipoprotein (LDL), triglycerides (TG), and total cholesterol (TC) were measured by double-antibody sandwich enzyme-linked immunosorbent assay using Beckman Coulter RT-6100.

### Statistical analysis:

All statistical analyses were made using SPSS22.0. The measurement data that met normal distribution were expressed as (*χ̅*±*S*). A single-factor analysis of variance was used to compare multiple groups, and an LSD test was used to compare two groups. Counting data were expressed as n (%), and the chi-squared test was used for comparison between groups. Pearson and Spearman correlations were used to test the correlation between variables. The receiver operating characteristic (ROC) curve was used to evaluate the predictive value of the CT L/S ratio for NAFLD patients with AP. *P*<0.05 indicated a statistically significant difference.

## RESULTS

**Fig.1:**
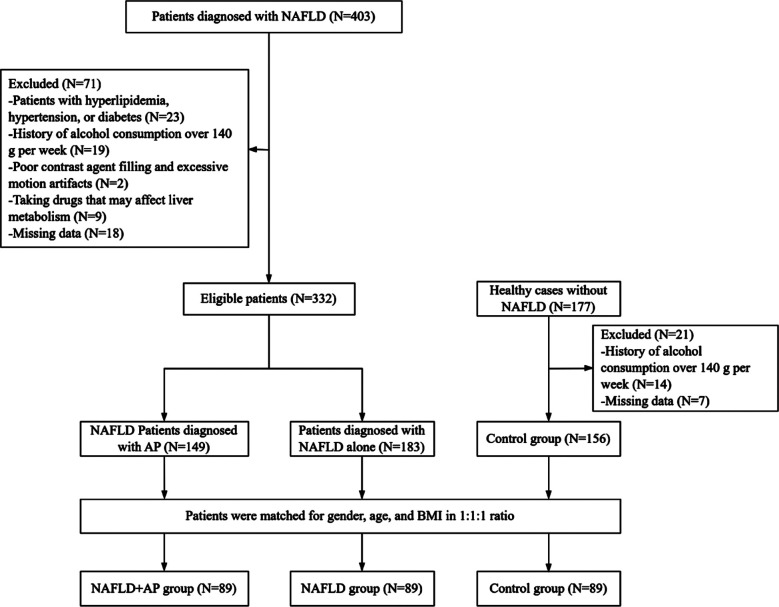
Flowchart of the study population.

**Fig.2:**
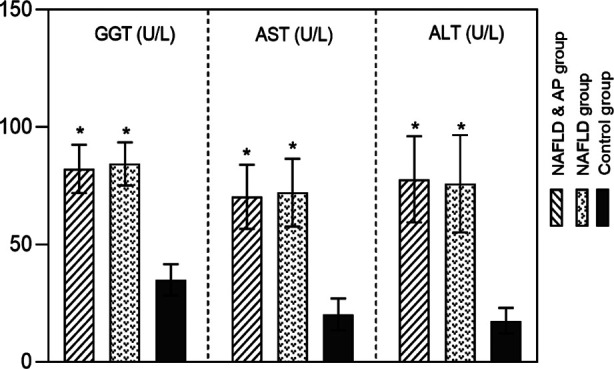
Comparison of liver function index levels among three groups; non-alcoholic fatty liver disease(NAFLD); atherosclerotic plaque (AP); gamma-glutamyl transferase (GGT), aspartate Transaminase(AST), and Alanine transaminase (ALT); Compared with the control group, *P<0.05; Compared to the NAFLD group, #P<0.05.

Eighty-nine cases in each group were matched for analysis (Fig.1). The NAFLD+AP group included 64 males and 25 females. The age of the patients ranged from 38 to 71 years, with a mean age of 53.53±9.74 years; body mass index (BMI) ranged from 18.55 to 26.61 kg/m^2^, with a mean BMI of 22.87±2.12 kg/m^2^. The NAFLD group included 61 males and 28 females. The age of the patients ranged from 36 to 73 years, with a mean age of 56.1±8.41 years; BMI ranged from 19.04 to 27.18 kg/m^2^, with a mean BMI of 23.16±2.27 kg/m^2^. The control group included 59 males and 30 females. The patients’ ages ranged from 37 to 74, with a mean age of 55.65±9.38 years; their BMI ranged from 18.23 to 27.70 kg/m^2^, with a mean BMI of 23.26±2.45 kg/m^2^. There were no significant differences in baseline characteristics such as age, sex, and BMI among the three groups (*P*>0.05) (Table-I). As shown in Fig.2, GGT, AST, and ALT levels in the NAFLD+AP and NAFLD groups were higher than those in the control group (*P*<0.05). GGT, AST, and ALT levels were comparable in all patients with NAFLD, regardless of the presence of an AP diagnosis (*P*>0.05).

**Table-I:** Baseline information.

Groups	N	Gender (male/female)	Age (year)	BMI (kg/m²)
NAFLD+AP group	89	64/25	53.53±9.74	22.87±2.12
NAFLD group	89	61/28	56.10±8.41	23.16±2.27
Control group	89	59/30	55.65±9.38	23.26±2.45
*F*		0.664	1.989	0.726
*P*		0.717	0.139	0.485

**Fig.3:**
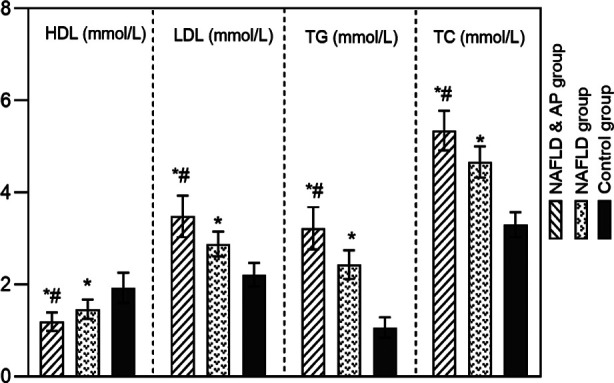
Comparison of blood lipid index levels among three groups; non-alcoholic fatty liver disease(NAFLD); atherosclerotic plaque (AP); High-density lipoprotein (HDL); Low density lipoprotein(LDL); triglycerides(TG); total cholesterol(TC); Compared with the control group, *P<0.05; Compared to the NAFLD group, #P<0.05.

**Fig.4:**
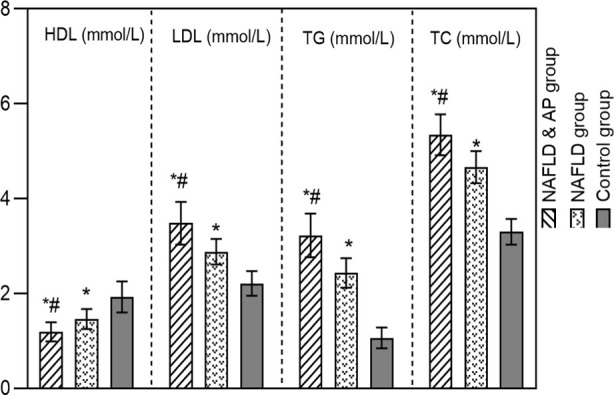
Comparison of CT L/S attenuation ratio in three groups; non-alcoholic fatty liver disease (NAFLD); atherosclerotic plaque (AP); Compared with the control group, # P#0.05; Compared to the NAFLD group, # P#0.05.

HDL levels in the NAFLD+AP and NAFLD groups were lower than in the control group, while the LDL, TG, and TC levels were higher (*P*<0.05). The HDL level of the NAFLD+AP group was lower than that of the NAFLD group, while the LDL, TG, and TC levels were higher than those of the NAFLD group (*P*<0.05) (Fig.3). The CT L/S attenuation ratio in all NAFLD patients was lower than in the control group (*P*<0.05), and significantly lower in the NAFLD+AP group compared to the NAFLD group (P<0.05) (Fig.4).

Pearson and Spearman’s tests confirmed that there was no significant correlation between the L/S attenuation ratio and liver function indicators GGT, AST, and ALT (*P*>0.05). The L/S attenuation ratio positively correlated with the levels of HDL, and negatively correlated with the levels of LDL, TG, and TC (*P*<0.05). Table-II.

**Table-II:** Correlation analysis of CT L/S attenuation ratio with liver function and blood lipid levels.

Index		GGT	AST	ALT	HDL	LDL	TG	TC
CT L/S attenuation ratio	*r*	-0.087	-0.097	0.016	0.422	-0.319	-0.381	-0.319
	*P*	0.416*	0.364*	0.882^#^	<0.001*	0.002^#^	<0.001*	0.002*

***Note:***

* indicates using Pearson test;

# indicates using Spearman test.

**Fig.5:**
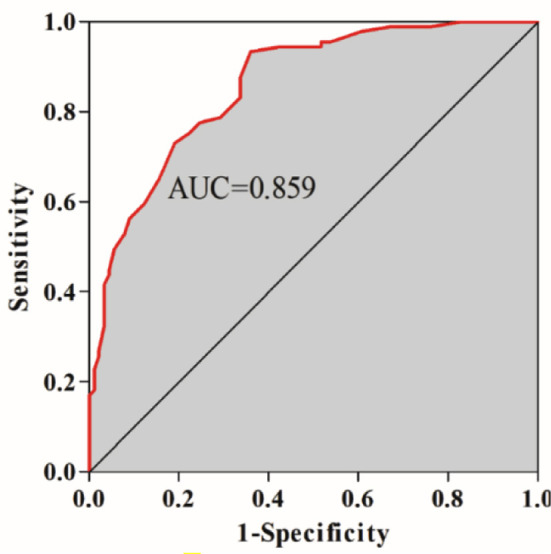
ROC Analysis.

The results of ROC analysis showed that the L/S attenuation ratio had a high predictive value for NAFLD patients with AP (AUC=0.859). According to the principle of maximum Youden’s J statistic, the optimal cut-off value was 0.59 (Fig.5 and Table-III).

**Table-III:** ROC analysis.

AUC	95%CI	P	Sensitivity(%)	Specificity(%)	Cut-off value
0.859	0.806-0.912	<0.001	93.3	64.0	0.59

ROC: receiver operating characteristic; AUC: area under the curve.

## DISCUSSION

This study found that the CT L/S attenuation ratio of the NAFLD+AP and NAFLD patients was significantly lower than in the healthy population. Moreover, NAFLD accompanied by AP was associated with a markedly lower L/S attenuation ratio than NAFLD alone. Our results indicate that while NAFLD led to a decrease in the L/S attenuation ratio, this decrease was more pronounced in patients who were also diagnosed with AP. The results of ROC analysis showed that the CT L/S attenuation ratio has a high predictive value for NAFLD+AP (AUC=0.859).

Our results are in agreement with previous studies. Shi W *et al*.^18^ demonstrated that the CT L/S attenuation ratio could be valuable for NAFLD screening of atherosclerotic plaques. A study by Yang *et al*.^19^ showed that in NAFLD patients, the decrease in cell oxidation due to fat infiltration of liver cells leads to degeneration and fibrosis of liver fat, resulting in liver hardening and a decrease in the L/S attenuation ratio, which is consistent with the conclusions of our study. In addition, Hideo Kajita et al.^20^ showed that compared to patients with NAFLD alone, NAFLD patients with accompanying diagnosis of AP present with liver hypoxia and ischemia due to obstruction of blood vessels. This leads to lower β-cell oxidation, further exacerbates liver fat fibrosis and degeneration, and manifests as an abnormal decrease in the L/S attenuation ratio.

Results of this study show that NAFLD was associated with elevated GGT, AST, and ALT levels regardless of AP diagnosis. This observation demonstrates that while liver function indicators can confirm the diagnosis of NAFLD, they have no clinical significance in predicting the occurrence of AP in this group of patients. It is plausible that excessive deposition of fat causes steatosis in liver cells and pathological damage to liver mitochondria and cell membranes, leading to abnormal increases in GGT, AST, and ALT levels. This is consistent with previous findings.^21^,^22^ Wojcik *et al*.^23^ pointed out that NAFLD patients often have elevated GGT, AST, and ALT levels. However, as some patients with severe NAFLD and NAFLD+AP may still exhibit normal levels of liver function indicators, there is no significant correlation between the levels of these indicators and the severity of NAFLD. In addition, while AST, ALT, and GGT can determine the degree of functional and structural liver damage, they are not useful predictors of atherosclerotic changes in the vascular system outside the liver tissue.

Heeren *et al*.^24^ showed that dyslipidemia is closely related to the onset and progression of NAFLD. This effect is mainly due to the disruption of the LDL/TG balance, which can lead to abnormal LDL synthesis and secretion, and eventually to NAFLD. The results of this study showed that NAFLD was associated with lower HDL and higher LDL, TG, and TC levels compared to reference values in the healthy population. We also reported significant differences in the blood lipid levels between the NAFLD+AP and NAFLD groups. Correlation analysis found that the L/S attenuation ratio positively correlated with HDL and negatively correlated with LDL, TG, and TC levels.

We may speculate that dyslipidemia can increase blood viscosity, affect blood flow, cause lipid deposition in the blood, and increase the risk of AP formation.^25^ Atherosclerotic changes, in turn, can cause abnormal vascular microcirculation, adversely affect oxygen and blood supply of the liver tissue, and aggravate steatosis. Moreover, lipid metabolism disorders and abnormal blood oxygen supply can reduce the generation of protective factors and affect LDL synthesis and storage in liver fat, leading to the formation of AP in patients with NAFLD.^26^ Liver dysfunction caused by NAFLD can exacerbate lipid metabolism disorders, thereby forming a vicious cycle.^25^,^26^

### Limitations:

First, it was a single-center retrospective study with a small sample size, which may have led to selection bias. Second, the CT indicators may have been influenced by human or technical factors, although the procedures were performed by the same team of experienced physicians. Higher-quality studies are needed to validate our conclusions.

## CONCLUSION

The CT L/S attenuation ratio in NAFLD patients with AP is significantly reduced and closely related to the levels of blood lipid indicators. The CT L/S attenuation ratio has a high predictive value for NAFLD with AP.

### Authors’ contributions:

**DH:** Conceived and designed the study and Review.

**DH**, **CH**, **SG**, **DZ** and **LX:** Collected the data and performed the analysis and Review.

**DH:** Was involved in the writing of the manuscript and is responsible for the integrity of the study.

All authors have read and approved the final manuscript.
